# Chromosome-level genome of the long-tailed marine-living ornate spiny lobster, *Panulirus ornatus*

**DOI:** 10.1038/s41597-024-03512-9

**Published:** 2024-06-22

**Authors:** Xianyun Ren, Dongfang Sun, Jianjian Lv, Baoquan Gao, Shaoting Jia, Xueqiong Bian, Kuangcheng Zhao, Jitao Li, Ping Liu, Jian Li

**Affiliations:** 1 National Key Laboratory of Mariculture Biobreeding and Sustainable Goods, Yellow Sea Fisheries Research Institute, Chinese Academy of Fishery Sciences, Qingdao, Shandong 266071 China; 2 Laboratory for Marine Fisheries Science and Food Production Processes, Laoshan Laboratory, Qingdao, Shandong 266237 China; 3 College of Fisheries and Life Science, Shanghai Ocean University, Shanghai, PR China

**Keywords:** Molecular evolution, Conservation biology

## Abstract

Recent conservation efforts to protect rare and endangered aquatic species have intensified. Nevertheless, the ornate spiny lobster (*Panulirus ornatus*), which is prevalent in the Indo-Pacific waters, has been largely ignored. In the absence of a detailed genomic reference, the conservation and population genetics of this crustacean are poorly understood. Here, We assembled a comprehensive chromosome-level genome for *P. ornatus*. This genome—among the most detailed for lobsters—spans 2.65 Gb with a contig N50 of 51.05 Mb, and 99.11% of the sequences with incorporated to 73 chromosomes. The ornate spiny lobster genome comprises 65.67% repeat sequences and 22,752 protein-coding genes with 99.20% of the genes functionally annotated. The assembly of the *P. ornatus* genome provides valuable insights into comparative crustacean genomics and endangered species conservation, and lays the groundwork for future research on the speciation, ecology, and evolution of the ornate spiny lobster.

## Background & Summary

Lobsters, with a prestigious status as valuable marine resources, are highly sought after in global fisheries for their economic and culinary significance. This has placed considerable focus on lobsters within the realms of biology, fisheries, and taxonomy^1^. The marine lobster family presently encompasses 49 acknowledged species, including 11 genera^2^. Lobsters, notable for their large size as benthic invertebrates, have exceptionally long lives, with some species estimated to live over 50 years and possibly up to 100 years^3^. However, the high market demand for lobsters resulted in intensive overfishing. Few countries have implemented effective management strategies to ensure sustainable harvests, and inadequate enforcement of fishing and marketing regulations have, in many regions, put significant strain on lobster populations. Consequently, to safeguard these valuable species and ensure their long-term sustainability, there is an urgent need to explore and implement alternative management approaches, such as co-management^4^.

**Fig. 1:**
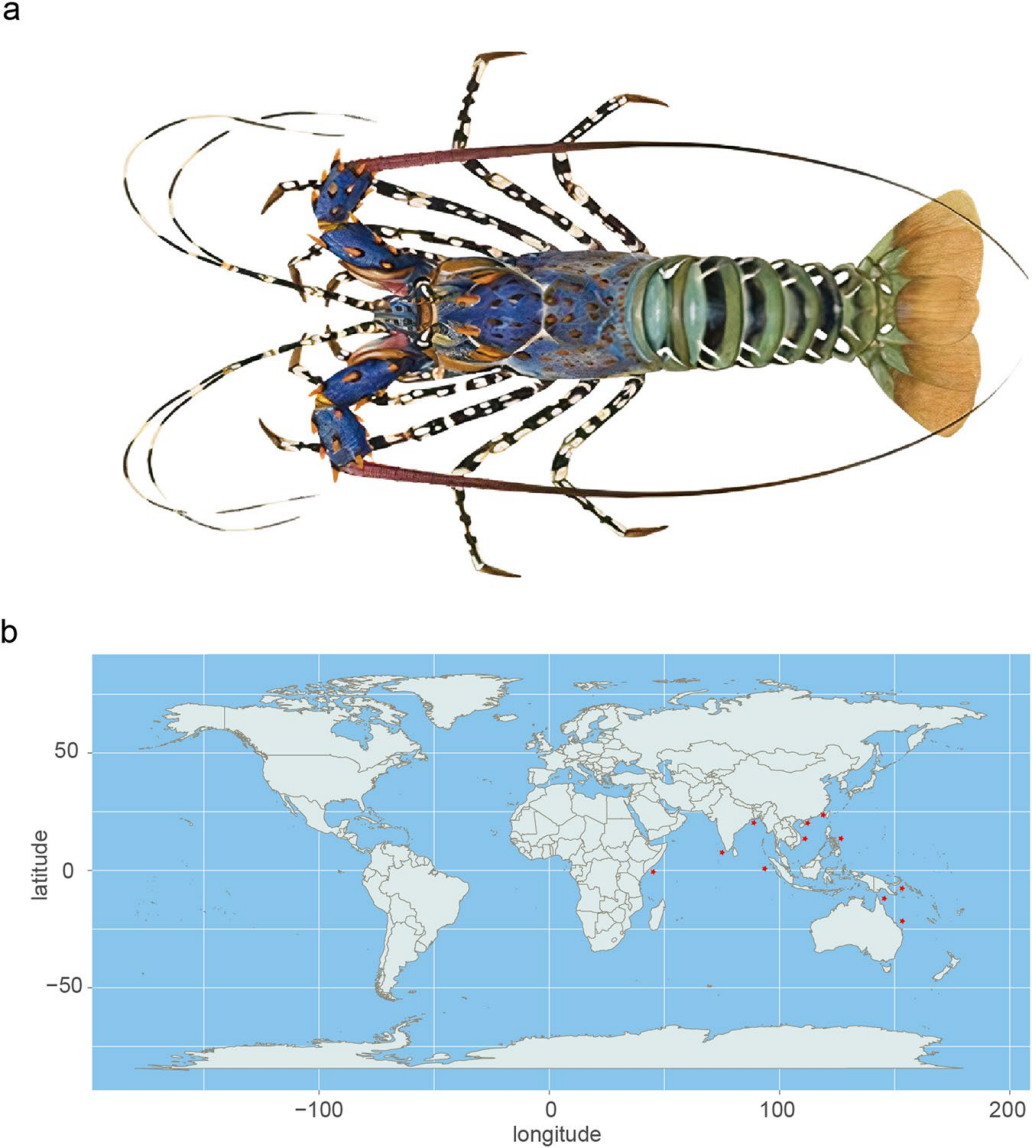
Photograph and location distribution of the long-tailed marine-living ornate spiny lobster, *Panulirus ornatus*. (**a**) A photo of the adult *P. ornatus*. (**b**) A natural distribution map of *P. ornatus* (red star).

The ornate spiny lobster, *Panulirus ornatus*, is an endangered species found on coral reefs and inshore habitats widely distributed in China, the South Pacific, and the Indian Ocean (Fig. 1a,b). In global aquaculture, it ranks as one of the most valued and highly priced fisheries^5^, and is consequently overexploited in unregulated fisheries^6,7^. On February 5, 2021, the ornate spiny lobster (*P. ornatus*) was classified as a Second Class species on China’s National Key Protected Wild Animals List—a notable conservation milestone, making *P. ornatus* the first crustacean to be recognized and included in this crucial protection list^8^. Like many other valued marine species around the globe, the ornate spiny lobster population faces several critical threats, including marine environmental pollution, injuries from fishing activities, loss of vital habitats, and a decline in fish resources^9^. The combined effects of global climate change and human activities exacerbate these challenges, posing significant risks to the survival and health of lobsters^10^. In conclusion, the population size of *P. ornatus* is in decline, and the pursuit of further conservation measures for these species is imperative.

**Fig. 2:**
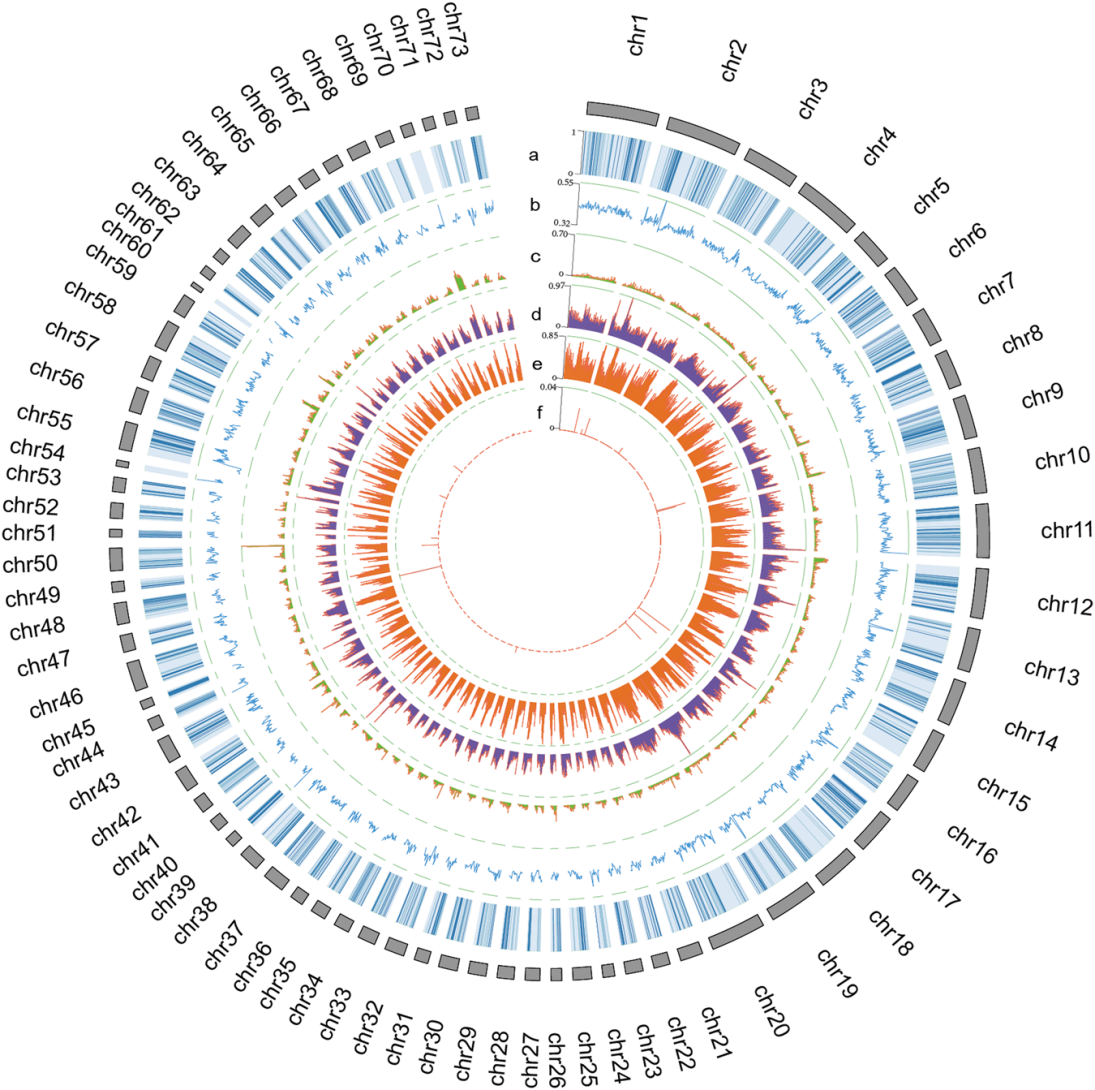
Genomic landscape of *P. ornatus*. Circos plot of *P. ornatus*. From outside to inside, gene density. (**a**), GC content (**b**), and the densities of DNA transposons (**c**), LTRs (**d**), LINEs (**e**), and SINEs (**f**), all represented in 200-kb genomic windows.

Previous attempts to sequence the genome of this species resulted in an incomplete and fragmented assembly, with an estimated genome size of 3.23 Gb compared to the actual assembled genome size of 1.93 Gb and a contig N50 of 5,451 bp, limiting the depth of potential research^11^. Here, we successfully achieved the first chromosome-level genome assembly for an endangered lobster species by integrating a combination of Illumina short reads, PacBio long read DNA sequencing, and Hi-C technology (Fig. 2). The project amassed 182.90 Gb of Illumina short-read data, 115.67 Gb of PacBio continuous long read data, and 456.71 Gb of Hi-C data, culminating in an assembled genome size of 2.65 Gb and a scaffold N50 of 51.05 Mb (Tables 1 and 2). Our high-quality genome assembly enhances the genomic resources available for crustaceans and provides essential data for their further protection.

**Table 1:** Statistics of the sequencing data.

Libary Types	Platform	Sample	Data size (Gb)	Average length (bp)	Sequencing Coverage (X)
WGS short reads	Illumina HiSeq 6000	Muscle	182.90	150	69
WGS long reads	Pacbio Sequel II	Muscle	292.02	–	110
Hi-C	Illumina HiSeq 6000	Muscle	456.71	150	172.34
RNA-seq	Illumina HiSeq 6000	Testis, Intestines, Hepatopancreas, Hemocytes, Muscle, Gills, Heart, Eyestalk	54.38	150	20

**Table 2:** Assembly statistics of the ornate spiny lobster.

	PacBio				Hi-C			
	Length		Number		Length		Number	
	Contig (bp)	Scaffold (bp)	Contig	Scaffold	Contig (bp)	Scaffold (bp)	Contig	Scaffold
Total	2,651,872,113	2,651,872,114	8,061	8,060	2,651,872,113	2,651,872,114	8,065	1,456
Max	26,555,445	26,555,445	–	–	26,555,445	106,421,940	–	–
Number ≥ 100Kb	2,470,197,054	2,470,197,054	2,162	2,162	2,470,197,054	2,633,032,281	2,162	92
N50	5,119,584	5,204,705	131	131	5,066,086	51,049,391	132	20
N90	160,956	160,956	1,501	1,500	160,500	19,445,244	1504	57

## Methods

### Sample collection and nucleic acid extraction

We collected male adult *P. ornatus* from Huangliu Co., LTD. in Sanya, Hainan, China. In this study, muscle tissue samples were collected and meticulously washed three times with sterile phosphate-buffered saline (PBS). The samples were then instantly froze with liquid nitrogen and subsequently stored at −80 °C. Total genomic DNA (gDNA) was extracted for genome survey and construction of the genome sequence libraries using the AMPure bead cleanup kit following the manufacturer’s instructions (Beckman Coulter, High Wycombe, UK). Meanwhile, we extracted total RNA from eight tissues (testis, intestines, hepatopancreas, hemocytes, muscle, gills, heart, and eyestalk) of the same individual by utilizing the TRIzol reagent according to the manufacturer’s instructions and subjected to RNA-seq analysis for genome structure annotation. The integrity and quality of the extracted nucleic acids were evaluated using 1.5% agarose gel electrophoresis and nucleic acid concentrations were accurately quantified using a Qubit fluorometer (Thermo Fisher Scientific based in Waltham, MA).

### Library construction and sequencing

A short-read library was prepared with an insert size of 350 bp and sequenced utilizing the Illumina Platform to generate 2 × 150 bp reads with NEB Next* Ultra^TM^ DNA Library Prep Kit (NEB, USA) for Illumina short-read sequencing following the manufacturer’s recommendations. For PacBio sequencing, we used genomic DNA to construct SMRTbell libraries following the manufacturer’s guidelines. We then sequenced the libraries using a PacBio Sequel platform equipped with single molecule real-time (SMRT). These sequencing efforts led to the generation of 182.90 Gb of Illumina short-read data and 292.02 Gb of raw continuous long reads (CLR), achieving a comprehensive 179-fold coverage of the *P. ornatus* genome (Table 1).

For Hi-C library construction, we used the MboI restriction enzyme to digest cross-linked high molecular weight (HMW) gDNA. After 5′ overhang biotinylation and blunt-end ligation, we physically sheared DNA into 300–500 bp fragments. Finally, we sequenced the Hi-C library with a strategy of 2 × 150 bp on the Illumina HiSeq using the NovaSeq 6000 platform, resulting in 456.71 Gb of paired-end raw reads. The sequencing libraries were then constructed using the NEBNext® UltraTM RNA Library Prep Kit for Illumina® (NEB, USA), with all procedures strictly adhering to the manufacturer’s recommendations. We then sequenced the RNA-seq library using the Illumina HiSeq 6000 platform to generate 2 × 150 bp reads. From this process, we generated 54.38 Gb of paired-end short clean reads, as we detail in Table 1.

### Genome survey and assembly

The adapter sequences and low-quality reads obtained from the Illumina platform were removed before the assembly process, using fastp software (version 0.23.1)^12^, retaining only clean reads for the subsequent stages of genome survey and assembly. We conducted genome surveys to determine key genomic characteristics such as overall size, heterozygosity, and repeatability, employing SOAPec (version 2.01)^13^ and GenomeScope (version 2.0)^14^ software to analyze 17 different K-mer frequencies. From these analyses, with a dominant peak depth of 59, we calculated the estimated genome size of *P. ornatus* to be 2917.34 Mb. We also approximated the heterozygosity and repetitive sequence content of the genome at 0.92% and 63.86%, respectively. In Table S1 and Fig. S1, we comprehensively detail these findings and estimates.

For genome assembly of *P. ornatus*, we employed a dual approach using two distinct assemblers—Wtdbg2 (version 2.5)^15^ and Flye (version 2.9)^16^—each of which produced an initial assembly using default parameters, which we then refined using the Arrow polishing process (version 8.0)^17^. Arrow is a consensus algorithm that generates highly accurate consensus sequences from PacBio subreads. After polishing, we merged the assemblies from Wtdbg2 and Flye using Quickmerge (version 0.3)^18^— a tool specifically designed to combine multiple genome assemblies into a single, unified consensus assembly. The resulting merged assembly was then polished twice using two rounds of Arrow and two rounds of Pilon (version 1.22)^19^ with default parameters. We performed PacBio subreads for Arrow and Illumina short reads for pilon, generating a total of 8,061 contigs with a total length of 2,651,872,113 bp (Table 2).

### Hi-C scaffolding

**Fig. 3:**
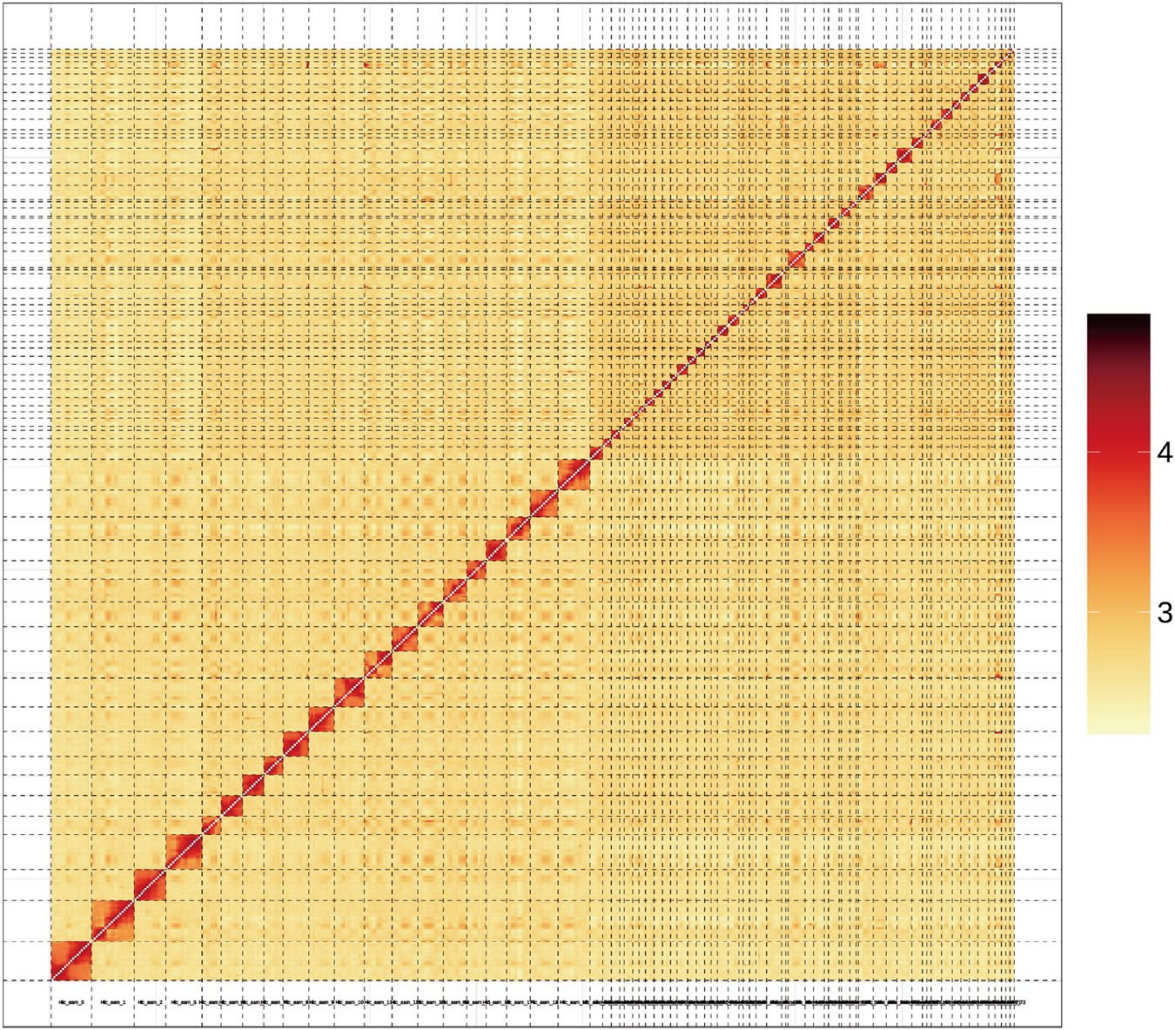
Hi-C heatmap (200-kb resolution) showcasing the interaction frequencies between different chromosomes of the ornate spiny lobster.

In the Hi-C scaffolding phase of this study, we first processed the raw Hi-C reads to eliminate adapters and low-quality bases, using fast software (version 0.23.1)^20^ with the parameters set to -q 20-l 50. Subsequently, we aligned these processed reads to the preliminary assembly using the Juicer pipeline^21^. Following alignment, we used the 3D-DNA pipeline^22^ to perform several critical tasks, including grouping the contigs into chromosomes, and orienting and ordering the contigs within each chromosome. To enhance the accuracy of the assembly, we manually corrected errors using Juicebox Assembly Tools (version 2.13.06)^21^. The scaffolding process allowed for accurate anchoring of 2,628.95 Mb of the assembly to 73 chromosomes (Fig. 3)—accounting for 99.11% of the total assembly (Table S2). The scaffold N50, a measure of assembly continuity, reached a length of 51.05 Mb in the final assembly (Table 2). This assembly is noteworthy for the contiguity of 14 chromosomes, each with no more than 30 gaps (Table 3).

**Table 3:** Assembly statistics for the chromosomes.

Name	Length (bp)	Gaps	Name	Length (bp)	Gaps
Chromosome 1	102,653,886	137	Chromosome 38	29,624,730	75
Chromosome 2	106,421,940	308	Chromosome 39	14,806,780	68
Chromosome 3	77,068,534	98	Chromosome 40	17,970,610	39
Chromosome 4	91,622,195	239	Chromosome 41	21,871,173	45
Chromosome 5	51,415,659	188	Chromosome 42	31,614,801	91
Chromosome 6	55,665,775	90	Chromosome 43	35,663,623	115
Chromosome 7	51,708,083	78	Chromosome 44	16,856,919	18
Chromosome 8	53,164,120	116	Chromosome 45	12,243,163	107
Chromosome 9	61,159,934	122	Chromosome 46	41,876,600	205
Chromosome 10	64,893,474	135	Chromosome 47	24,287,814	43
Chromosome 11	77,196,817	134	Chromosome 48	30,773,430	59
Chromosome 12	71,406,443	295	Chromosome 49	14,828,202	17
Chromosome 13	62,016,665	191	Chromosome 50	32,424,604	24
Chromosome 14	64,676,935	322	Chromosome 51	11,180,326	22
Chromosome 15	59,343,222	273	Chromosome 52	22,152,074	54
Chromosome 16	51,049,391	189	Chromosome 53	20,786,171	22
Chromosome 17	57,652,817	75	Chromosome 54	8,659,570	103
Chromosome 18	60,297,985	185	Chromosome 55	40,279,970	150
Chromosome 19	70,525,098	206	Chromosome 56	35,263,926	49
Chromosome 20	79,306,802	137	Chromosome 57	32,246,318	44
Chromosome 21	32,646,996	21	Chromosome 58	40,162,454	147
Chromosome 22	23,682,639	21	Chromosome 59	27,863,821	19
Chromosome 23	25,105,649	30	Chromosome 60	6,534,551	78
Chromosome 24	17,009,692	14	Chromosome 61	13,596,784	102
Chromosome 25	26,985,483	91	Chromosome 62	15,864,029	34
Chromosome 26	15,594,842	53	Chromosome 63	29,434,284	77
Chromosome 27	21,159,578	45	Chromosome 64	28,623,056	57
Chromosome 28	24,208,021	16	Chromosome 65	27,396,681	46
Chromosome 29	25,808,952	31	Chromosome 66	25,136,672	47
Chromosome 30	27,173,289	28	Chromosome 67	23,758,603	33
Chromosome 31	20,103,365	42	Chromosome 68	28,540,147	89
Chromosome 32	28,561,403	48	Chromosome 69	22,336,085	79
Chromosome 33	25,435,984	66	Chromosome 70	16,587,605	203
Chromosome 34	22,682,860	18	Chromosome 71	16,220,192	62
Chromosome 35	22,286,449	57	Chromosome 72	15,657,606	39
Chromosome 36	19,445,244	27	Chromosome 73	17,029,672	50
Chromosome 37	31,662,036	43			

**Fig. 4:**
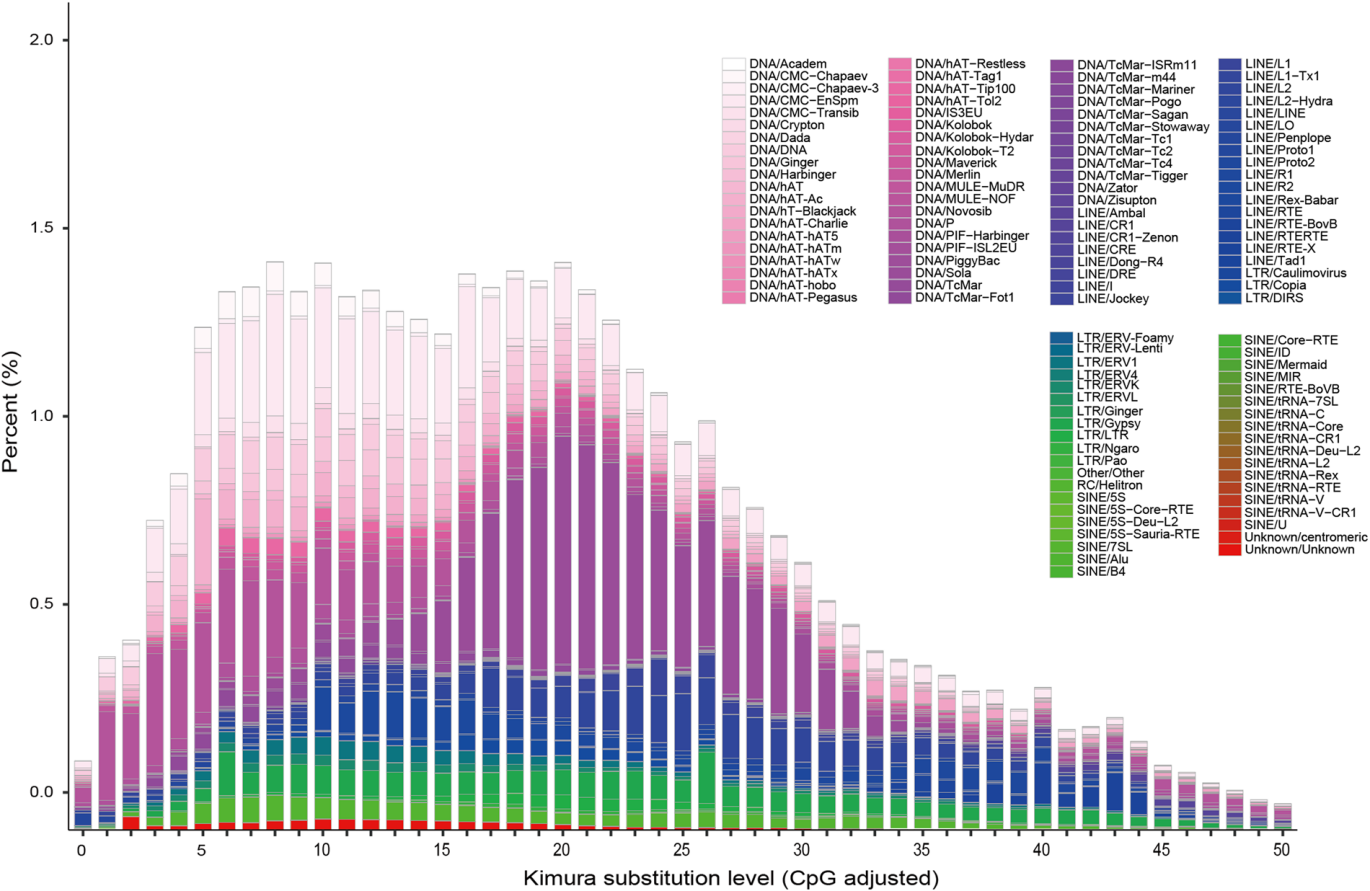
Distribution of divergence rates for TEs in the *P. ornatus* genome.

**Genomic repeat annotation**. We identified repeat sequences in the *P. ornatus* genome using both homology-based and *de novo* strategies^23^. Initially, we merged the *de novo* predicted repetitive sequence database with the Repbase homologous repetitive sequence database^24^. We used a suite of tools—RepeatScout (version 1.0.5)^23^, RepeatModeler (version 2.0.1)^25^, Piler (version 1.0)^26^, and LTR-FINDER (version 1.0.6)^27^—to identify transposable element (TE) families, whereafter we employed Repeatmasker (version 4.1.0)^25^, RepeatProteinMask (version 4.1.0), and TRF (version 4.0.9)^28^ to classify different repetitive elements. We achieved this classification by aligning the *P. ornatus* genome sequences with the integrated database. After eliminating redundant results from these three methods, we established that repeat sequences constituted 65.67% of the *P. ornatus* genome (Table S3). In addition, we calculated the Kimura divergence value of TEs using the script ‘calcDivergenceFromalign.pl^29^ and created TE landscapes with ‘createRepeatLandscape.pl^30^. Among the identified repeat elements, we identified DNA elements as comprising 4.58% of the genome, with long interspersed nuclear elements (LINEs) accounting for 40.30%. Short interspersed nuclear elements (SINEs) and long terminal repeats (LTRs) constituted only 0.01% and 30.07% of the genome, respectively (Table 4 and Fig. 4).

**Table 4:** Classification of repetitive sequences in the *P. ornatus* genome.

Type	Denovo + Repbase		TE proteins		Combined TEs	
	Length (bp)	% in Genome	Length (bp)	% in Genome	Length (bp)	% in Genome
DNA	114,614,205	4.32	7,546,890	0.28	121,591,493	4.58
LINE	815,534,599	30.75	598,598,242	22.57	1,069,071,691	40.30
SINE	353,960	0.01	0.00	0.00	353,960	0.01
LTR	793,326,193	29.91	7,844,049	0.30	797,496,967	30.07
Other	11,537	0.00	0.00	0.00	11,537	0.00
Unknown	6,362,387	0.24	0.00	0.00	6,362,387	0.24
Total	1,499,560,266	56.53	613,875,238	23.14	1,519,235,231	57.27

In the process of annotating noncoding RNA (ncRNA) within the *P. ornatus* genome, we employed specific tools for different types of ncRNA predictions. For tRNA prediction, we used tRNAScan (version 1.4)^31^, whereas for rRNA prediction we used Blast (version 2.2.26)^32^. To identify other types of ncRNAs, such as miRNA and snRNA, we aligned the sequences to the Rfam database^33^ using the INFERNAL tool (version 1.0)^34^. Using these methods, we successfully identified four distinct types of noncoding RNAs in the *P. ornatus* genome. including 12,771 miRNAs, 5,187 tRNAs, 1,716 rRNAs, and 1,296 snRNAs (Table 5).

**Table 5:** Classification of ncRNAs in the *P. ornatus* genome.

nc RNA type		Copy (w*)	Average length (bp)	Total length (bp)	Proportion in Genome (%)
miRNA		12,771	155.16	1,981,608	0.074706
tRNA		5,187	73.53	381,395	0.014379
rRNA	rRNA	858	90.8	77,904	0.002937
	18 S	343	116.19	39,853	0.001502
	28 S	5	105.6	528	0.00002
	5.8 S	0	0	0	0
	5 S	510	73.57	37,523	0.001415
snRNA	snRNA	659	245.24	161,613	0.006093
	CD-box	15	113.93	1,709	0.000064
	HACA-box	472	286.44	135,200	0.005097
	splicing	150	131.71	19,756	0.000745

### Protein-coding gene prediction and annotation

**Fig. 5:**
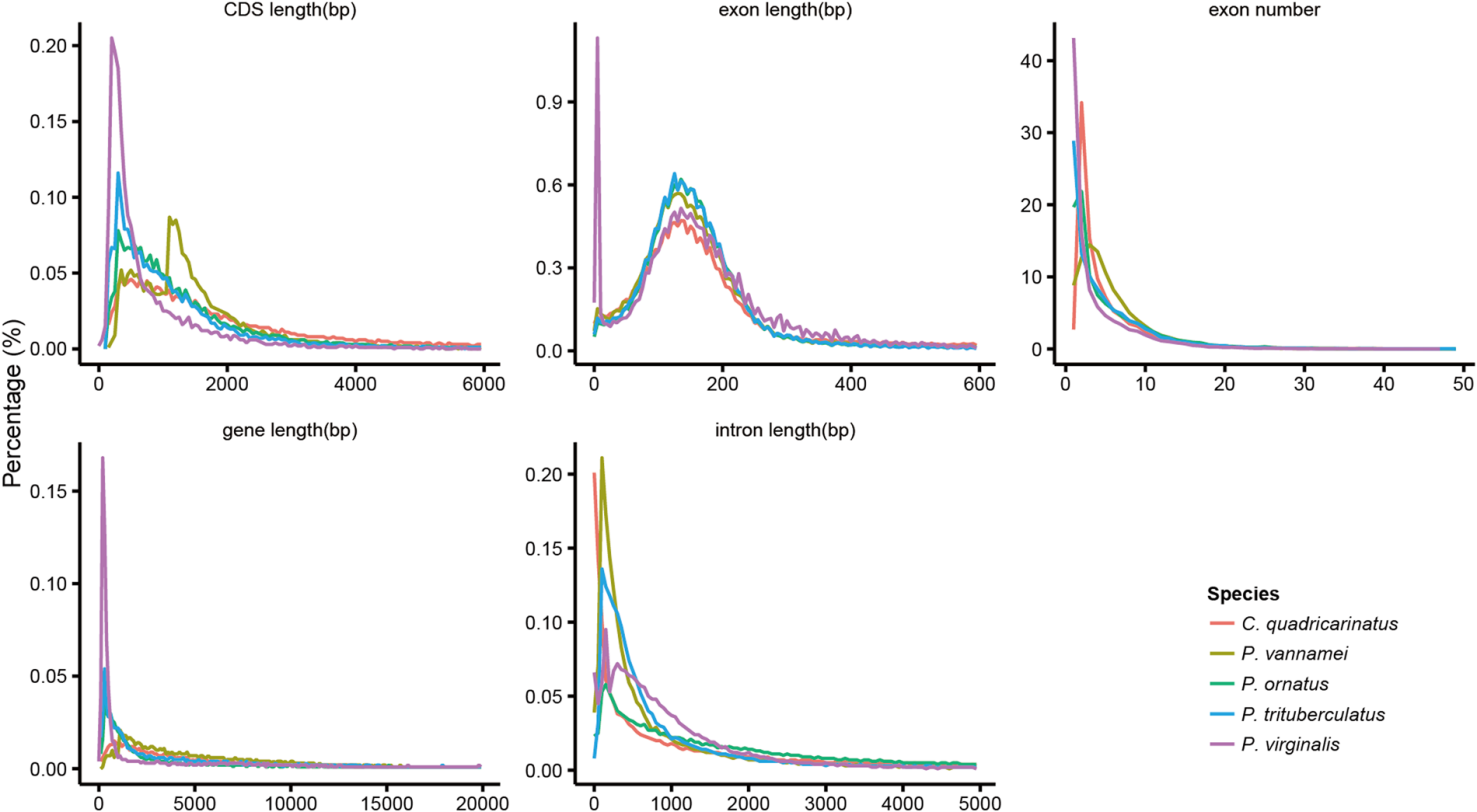
Comparisons of genomic elements of closely related species.

For gene structure prediction of the *P. ornatus* genome, we employed a combination of *de novo*, homology-based, and transcriptome sequencing-based predictions. For the *de novo* approach, we used a suite of tools–Augustus (v3.2.3)^35^, GlimmerHMM (v3.02)^36^, SNAP (v2013.11.29)^37^, Geneid (v1.4)^38^, and Genscan (v1.0)^39^—to predict gene structures directly from the genome sequence. For homologous-based annotation, the protein sequences of *Portunus trituberculatus* (swimming crab), *Cherax quadricarinatus* (Australian red claw crayfish), *Penaeus vannamei* (Pacific white shrimp), *Procambarus virginalis* (marbled crayfish), *Homo sapiens* (human), *Drosophila melanogaster* (fruit fly), *Tribolium castaneum* (red flour beetle), *Caenorhabditis elegans* (nematode), and *Crassostrea gigas* (Pacific oyster) were downloaded from the NCBI’s Genbank database, and aligned against spiny lobster genome using Blast (v2.2.26)^32^ and Genewise (v2.4.1)^40^. With this multifaceted approach, we ensured a thorough and accurate prediction of the protein-coding genes in the *P. ornatus* genome, thereby enhancing our understanding of its genetic architecture. We identify a total of 5,087–58,220 homolgous genes when comparing against the nine target species (Table 6) (Table 6). We analyzed the lengths of genes, CDS, exons, and introns in *P. ornatus* and compared them with those of five other species (Fig. 5). We found the average lengths for *P. ornatus* to be 29,875.91 bp for transcripts, 1,420.49 bp for CDS, 257.65 bp for exons, and 6,300.84 bp for introns (Table S4).

**Table 6:** Statistical analyses of gene structure annotation of the *P. ornatus* genome.

	Gene set	Number	Average transcript length (bp)	Average CDS length (bp)	Average exons per gene	Average exon length (bp)	Average intron length (bp)
*De novo*	Augustus	140,919	8,393.44	746.73	2.41	309.24	5,405.21
	GlimmerHMM	1,112,796	1,897.29	368.12	1.98	185.53	1,553.73
	SNAP	404,703	3,729.98	432.32	2.31	187.13	2,516.88
	Geneid	73,799	19,378.32	726.23	3.98	182.69	6,269.15
	Genscan	71,486	24,804.11	1,333.45	5.56	239.86	5,147.88
Homolog	*C. elegans*	5,087	11,249.79	801.12	3.16	253.74	4,843.48
	*C. gigas*	54,123	3,299.97	739.18	1.61	458.32	4,178.79
	*C. quadricarinatus*	2,428	4,567.96	889.1	1.8	494.44	4,609.01
	*D. melanogaster*	8,273	15,511.86	984.4	3.85	255.5	5,092.40
	*H. sapiens*	17,322	7,755.98	812.71	2.44	332.49	4,807.20
	*L. vannamei*	58,220	4,292.73	1,061.47	1.88	565.89	3,689.72
	*P. trituberculatus*	33,962	9,676.67	822.44	2.8	293.5	4,913.12
	*P. virginalist*	46,224	4,321.99	709.96	1.86	380.74	4,177.30
	*T. castaneum*	26,872	5,179.68	821.65	2.04	402.8	4,190.99
RNAseq	PASA	33,503	61,329.43	1,557.88	7.52	207.3	9,174.33
	Cufflinks	49,992	62,526.77	3,773.38	7.59	497.31	8,918.79
EVM		38,194	14,920.34	1,182.39	3.66	322.79	5,158.86
Pasa-update		37,841	19,224.22	1,228.87	3.86	318.31	6,290.64
Final set		22,752	29,857.91	1,420.49	5.51	257.65	6,300.84

Denovo: gene structure prediction using Augustus, GlimmerHMM, SNAP, Geneid and Genscan software; RNAseq: annotation of gene structures using transcriptome data; PASA is the result of Trinty assembly results predicted in combination with the transcriptome; Transcripts is the result of transcriptome prediction in combination with the reference genome; EVM: the result of integrating *Denovo* annotation, homology annotation, and RNA annotation results; Pasa-update: the result of correction based on PASA; Final set: the final result of functional gene annotation. Homolog set number indicates the number of orthologs genes found

**Fig. 6:**
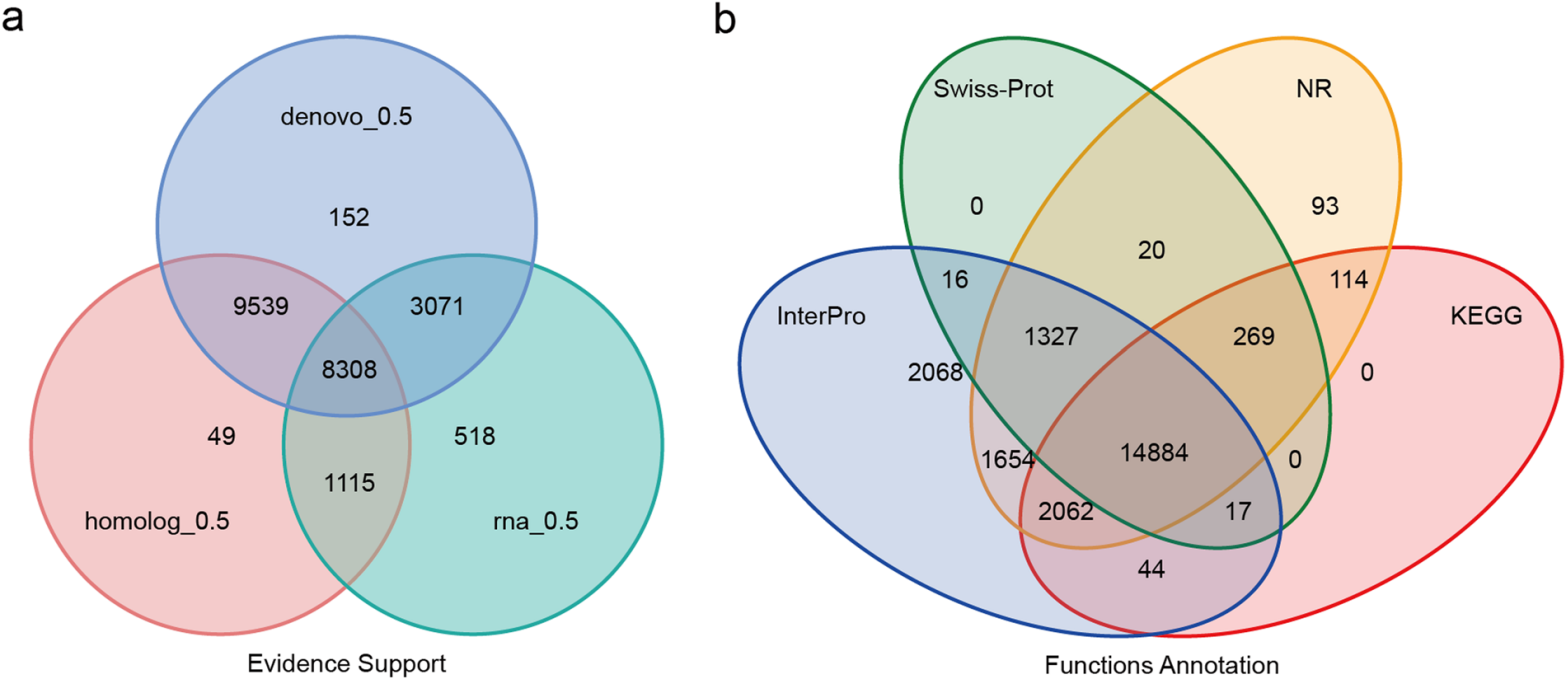
Gene prediction and functional annotation of the *P. ornatus* genome. (**a**) Venn diagram of the gene set prediction. (**b**) Venn diagram of functional annotation based on different databases.

Two assembly methods including transcript assembly with reference to the genome and *de novo* assembly using Trinity software (version 2.11.0)^41^ were utilized to process clean RNA-seq data. Open reading frames (ORFs) were identified using PASA (version 2.1.0)^42^, and gene sets predicted by the different methods were merged into a comprehensive, non-redundant gene set containing 22,752 protein-coding genes with Evidence Modeler (version 1.1.1)^43^ (Table 7 and Fig. 6a).

**Table 7:** Statistical analysis of functional gene annotations of the *P. ornatus* genome.

	Number	Percent (%)
Total	22,752	—
Swissprot	16,533	72.7
Nr	20,423	89.8
KEGG	17,390	76.4
InterPro	22,072	97
GO	19,598	86.1
Pfam	12,235	53.8
Annotated	22,568	99.2
Unannotated	184	0.8

We functionally annotated the protein-coding genes using Blastp (version 2.2.26)^44^ and Diamond (version 0.8.22)^45^ to align the genes against several protein databases, including SwissProt^46^, NCBI Nonredundant protein (NR), KEGG^47^, InterPro^48^, GO Ontology (GO)^49^, and Pfam^50^, setting the E-value cutoff at 1E-5. We further annotated protein domains and motifs using InterProScan (version 5.52–86.0)^51^. We annotated 22,568 (99.20%) of the 22,752 predicted genes, by at least one of these databases (Table 7). All four databases supported 14,884, or 65.42% of these functionally annotated proteins (Fig. 6b).

## Data Records

We deposited the genomic Illumina sequencing data in the SRA at NCBI SRR26801482^52^ and SRR26801483^53^.

We deposited the genomic PacBio sequencing data in the SRA at NCBI SRR26801477^54^ and SRR26801478^55^.

We deposited the transcriptomic sequencing data in the SRA at NCBI SRR SRR26945899^56^-SRR26945906^57–63^.

We deposited the Hi-C sequencing data in the SRA at NCBI SRR26801479–SRR 26801481^64–66^.

This Whole Genome Shotgun project has been deposited at GenBank under the accession https://identifiers.org/ncbi/insdc.gca:GCA_036320965.1^67^. The version described in this paper is version ASM3632096v1. The final chromosome assembly and genome annotation files are also available in Figshare^68^.

## Technical Validation

### Evaluation of genome assembly and annotation

We rigorously evaluated the quality of *P. ornatus* genome assembly using multiple methods. First, with the Benchmarking Universal Single-Copy Orthologs (BUSCO) (version 3.0.2)^69^ assessment, using the BUSCO database (arthropoda_odb9) of single-copy orthologous genes along with tools such as tblastn, augustus, and hmmer, we confirmed the presence of 93.6% of gene orthologs in *P. ornatus*, with 93.6% being complete and 3.2% fragmented, indicating a comprehensive assembly (Table S5). Second, employing the Core Eukaryotic Genes Mapping Approach (CEGMA) (version 2.5)^70^, we revealed that *P. ornatus* genes had homologs for 226 highly conserved core genes, accounting for 91.13% (248) of the total, further confirming the completeness of the assembly (Table S6). Finally, we aligned Illumina sequencing reads to the nuclear genome using BWA (version 0.7.8)^71^, resulting in a high read mapping rate of 97.85% and a coverage rate of 96.80%, demonstrating the better integrity of the assembled genome as well as the homogeneity of the sequencing data (Table S7). These collective findings indicate the high quality of *P. ornatus* genome assembly.

#### Collinearity analysis

**Fig. 7:**
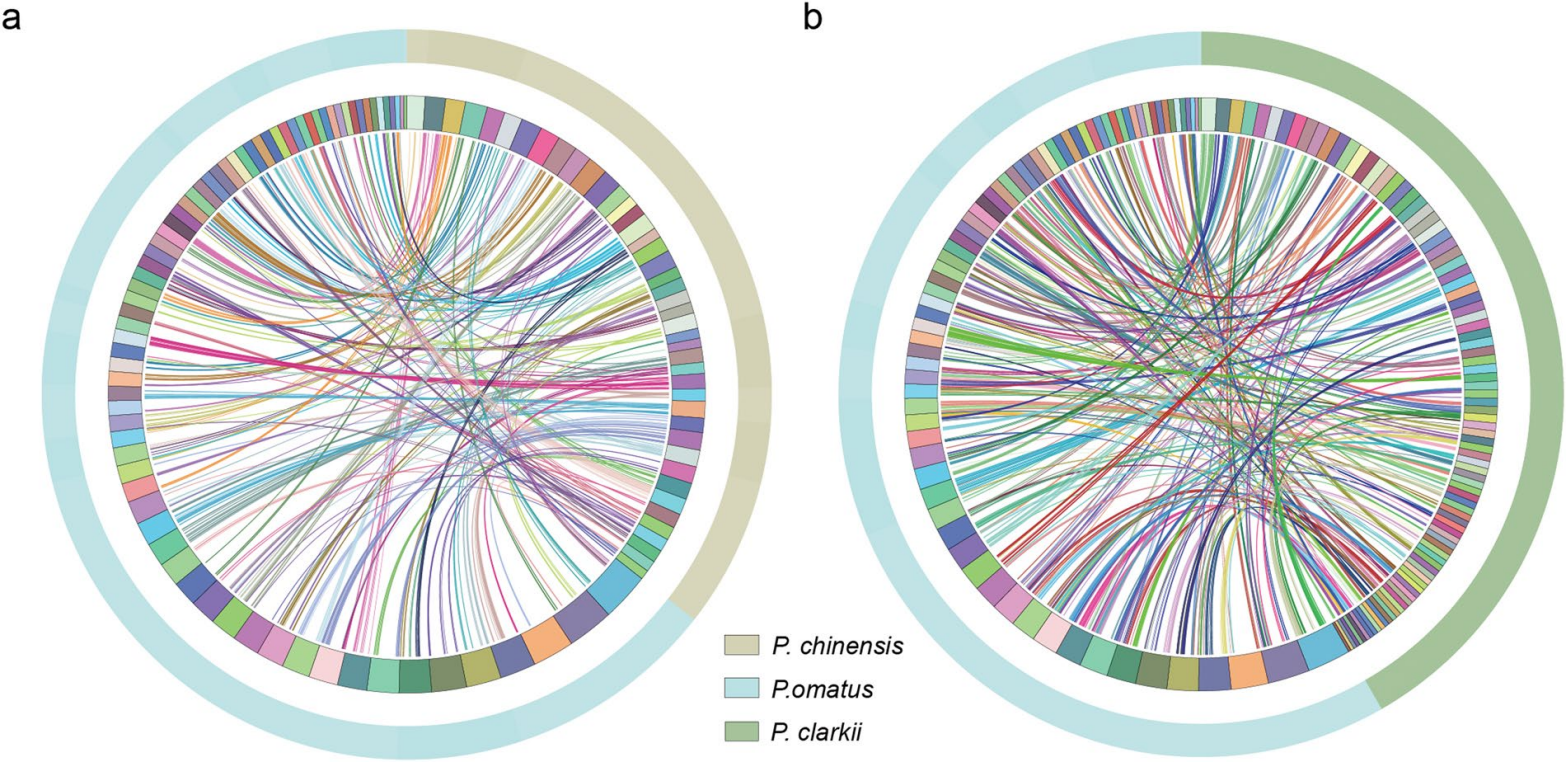
Chromosome sequence synteny comparisons. (**a**) Syntenic relationship between the *P. ornatus* genome and the *P. chinensis* genome. (**b**) Syntenic relationship between the *P. ornatus* genome and the *P. clarkii* genome. Each line connects a pair of homologous sequences between the two species.

For whole genome synteny comparison, we aligned the chromosome-level genomes of two decapod species, *Penaeus chinensis* and *Procambarus clarkii*, with the *P. ornatus* genome assembly, using LASTZ (version 1.02.00)^72^ with default parameters. We found that nearly 73 chromosome-level scaffolds of *P. ornatus* exhibited significant similarity with the corresponding chromosomes of *P. chinensis* and *P. clarkii* (Fig. 7). This similarity underscores the high quality of the sequencing and assembly of the *P. ornatus* genome, while improving the reliability of phylogenetic analyses.

In conclusion, we successfully assembled a high-quality chromosome-level genome of *P. ornatus*. This newly generated reference genome represents a significant contribution to our knowledge of lobster genetic diversity. It will not only advance comparative evolutionary studies but also play a crucial role in conservation efforts for this endangered species.

### Supplementary information

Figure S1, Figure S2

Table S1, Table S2, Table S3, Table S4, Table S5, Table S6, Table S7

## Data Availability

We detail all commands and pipelines employed for data processing in the methods section. For any software where specific parameters were not mentioned, we used the default settings recommended by the software developers. The core code is available at https://github.com/sundongfang/Chromosome-level-genome-of-Panulirus-ornatus.
