# Supplementary material for: Chromosome-level genome of the long-tailed marine-living ornate spiny lobster, Panulirus ornatus
Source: Sci Data. 2024 Jun 22;11:662. doi: 10.1038/s41597-024-03512-9 (PMC11193758; doi:10.1038/s41597-024-03512-9)

Contents

Figure S1. Distribution profiles of 17-mer analysis of Illumina reads......................... 2

Figure S2. The distribution of genes in different species. The horizontal axis represents 13 species, and the vertical axis represents the number of genes................ 3

Figure S1. Distribution profiles of 17-mer analysis of Illumina reads.

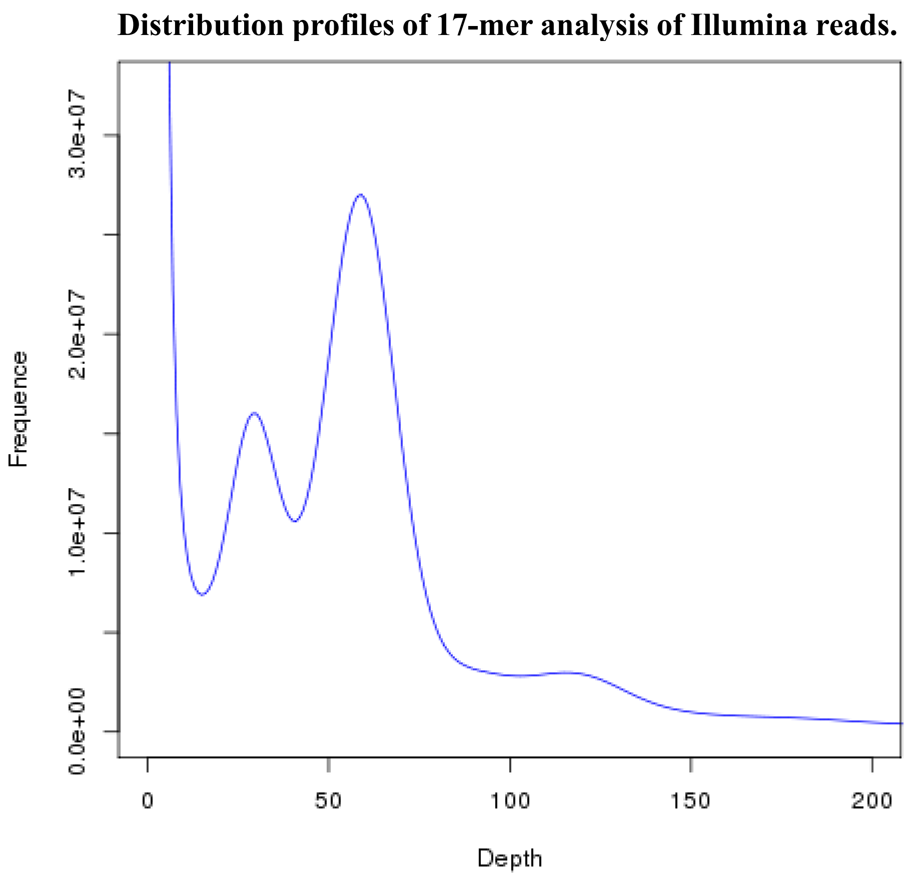

Figure S2. The distribution of genes in different species. The horizontal axis represents13 species, and the vertical axis represents the number of genes.

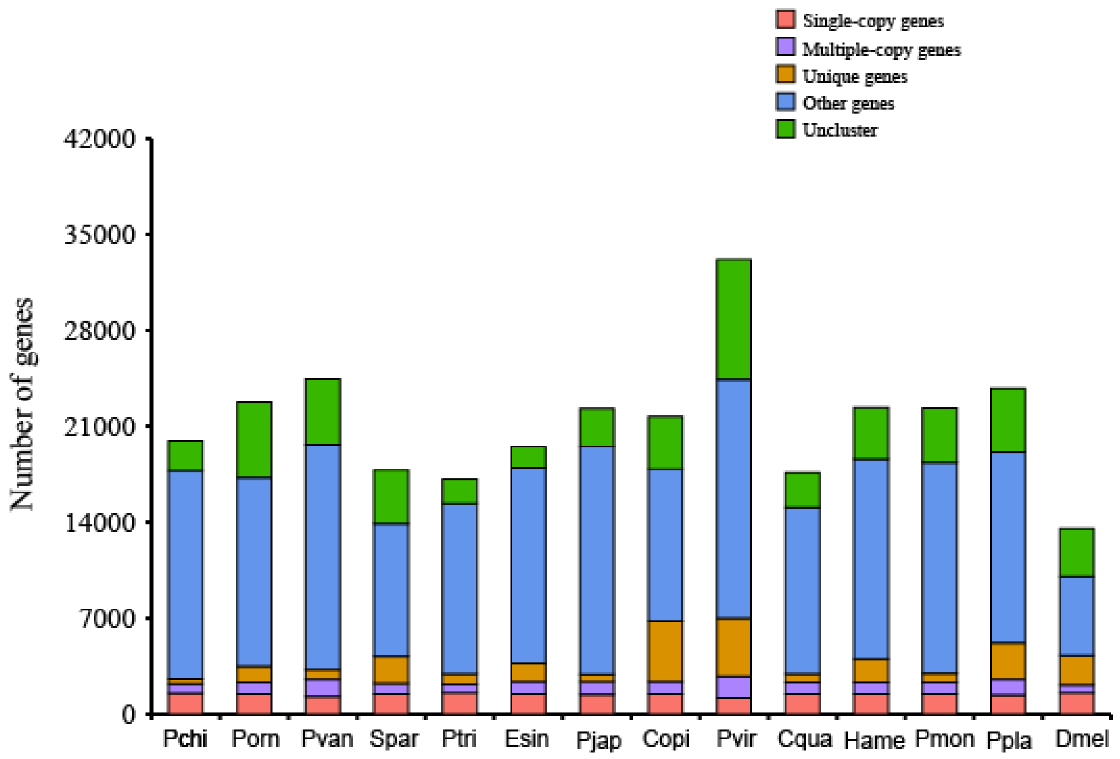

Supplement: Supplementary file 1 — Figure S1, Figure S2 [file 41597_2024_3512_MOESM1_ESM.pdf]
